# Antibiotic use and resistance in emerging economies: a situation analysis for Viet Nam

**DOI:** 10.1186/1471-2458-13-1158

**Published:** 2013-12-10

**Authors:** Kinh Van Nguyen, Nga Thuy Thi Do, Arjun Chandna, Trung Vu Nguyen, Ca Van Pham, Phuong Mai Doan, An Quoc Nguyen, Chuc Kim Thi Nguyen, Mattias Larsson, Socorro Escalante, Babatunde Olowokure, Ramanan Laxminarayan, Hellen Gelband, Peter Horby, Ha Bich Thi Ngo, Mai Thanh Hoang, Jeremy Farrar, Tran Tinh Hien, Heiman FL Wertheim

**Affiliations:** 1 National Hospital of Tropical Diseases, Hanoi, Viet Nam; 2 Oxford University Clinical Research Unit, Hanoi, Viet Nam; 3 Bach Mai Hospital, Hanoi, Viet Nam; 4 Ministry of Agriculture and Rural Development, Hanoi, Viet Nam; 5 Hanoi Medical University, Hanoi, Viet Nam; 6 World Health Organization, Country Office, Hanoi, Viet Nam; 7 Nuffield Department of Medicine, Centre for Tropical Medicine, University of Oxford, Oxford, UK; 8 Medical Service Administration, Ministry of Health, Hanoi, Viet Nam; 9 Drug Administration of Viet Nam, Ministry of Health, Hanoi, Viet Nam; 10 Hospital of Tropical Diseases, Ho Chi Minh City, Viet Nam

**Keywords:** Antibiotic resistance, Bacterial diseases, Health policy, Health systems, Legislation (health), Resource constrained, Antibiotic consumption

## Abstract

**Background:**

Antimicrobial resistance is a major contemporary public health threat. Strategies to contain antimicrobial resistance have been comprehensively set forth, however in developing countries where the need for effective antimicrobials is greatest implementation has proved problematic. A better understanding of patterns and determinants of antibiotic use and resistance in emerging economies may permit more appropriately targeted interventions.

Viet Nam, with a large population, high burden of infectious disease and relatively unrestricted access to medication, is an excellent case study of the difficulties faced by emerging economies in controlling antimicrobial resistance.

**Methods:**

Our working group conducted a situation analysis of the current patterns and determinants of antibiotic use and resistance in Viet Nam. International publications and local reports published between 1-1-1990 and 31-8-2012 were reviewed. All stakeholders analyzed the findings at a policy workshop and feasible recommendations were suggested to improve antibiotic use in Viet Nam.

Here we report the results of our situation analysis focusing on: the healthcare system, drug regulation and supply; antibiotic resistance and infection control; and agricultural antibiotic use.

**Results:**

Market reforms have improved healthcare access in Viet Nam and contributed to better health outcomes. However, increased accessibility has been accompanied by injudicious antibiotic use in hospitals and the community, with predictable escalation in bacterial resistance. Prescribing practices are poor and self-medication is common – often being the most affordable way to access healthcare. Many policies exist to regulate antibiotic use but enforcement is insufficient or lacking.

Pneumococcal penicillin-resistance rates are the highest in Asia and carbapenem-resistant bacteria (notably NDM-1) have recently emerged. Hospital acquired infections, predominantly with multi-drug resistant Gram-negative organisms, place additional strain on limited resources. Widespread agricultural antibiotic use further propagates antimicrobial resistance.

**Conclusions:**

Future legislation regarding antibiotic access must alter incentives for purchasers and providers and ensure effective enforcement. The Ministry of Health recently initiated a national action plan and approved a multicenter health improvement project to strengthen national capacity for antimicrobial stewardship in Viet Nam. This analysis provided important input to these initiatives. Our methodologies and findings may be of use to others across the world tackling the growing threat of antibiotic resistance.

## Background

Antimicrobial resistance is a global concern and a particularly pressing issue in resource-limited countries. Respiratory, diarrheal, sexually-transmitted and nosocomial infections are leading causes of death in the developing world [1] and their management is critically compromised by the appearance and rapid spread of resistance.

Antibiotic drug pressure is a key driver of resistance. Whilst it is an unavoidable consequence of antibiotic use – both rational and irrational – unnecessary antibiotic pressure can and must be reduced. The World Health Organization (WHO) published comprehensive recommendations designed to restrict the emergence and spread of antimicrobial resistant organisms, promoting prudent use of antimicrobials in humans, food-producing animals and aquaculture [2].

With a population exceeding 91 million, Viet Nam is the world’s 13th most populous country (Table 1) and has a rapidly developing economy [3,4]. In 1986 a series of economic reforms, termed the Đổi Mới, facilitated the transition to a more market-driven economy. One of the consequences of economic liberalization has been a relatively unregulated access to antimicrobials and this, coupled with the high burden of infectious disease, has made Viet Nam a potential hotspot for the emergence of drug resistance.

**Table 1:** Key health and development indicators in Viet Nam

**Indicator**	**Year**	**Number**
Population (millions)	2013	92.48
Population growth rate (%)	2013	1.03
Urbanization rate (%)	2010-15	3.03
Life expectancy (male/female)	2010-15	77.4/73.4
GDP per capita (PPP) (US$)	2012	3,600
Infant mortality rate (per 1000)	2010-15	18.3
Maternal mortality ratio (per 100,000 live births)	2010	59
Poverty rate (% < 1.25USD/day)	2012	11.3
Access to improved drinking water sources (%)	2010	95
Access to improved sanitation facilities (%)	2010	76
Adult literacy rate (%)	2011	93.4

Sources: World Health Organization (http://apps.who.int/gho/data/view.country.21300, date accessed: 09/10/2013).

United Nations (http://data.un.org/CountryProfile.aspx?crName=Viet%20Nam, date accessed: 09/10/2013).

CIA World Factbook (https://www.cia.gov/library/publications/the-world-factbook/geos/vm.html, date accessed: 09/10/2013).

Our paper presents a situation analysis that addresses the current patterns and determinants of antibiotic use and resistance in Viet Nam. It suggests how the information gained during this process can be used to develop effective interventions to improve antibiotic stewardship in Viet Nam, whilst taking care not to diminish access to these life-saving drugs.

Formulating policy to improve antibiotic stewardship is best accomplished by beginning with a broad analysis of a country and its health system. The first iteration of such an analysis was produced for Viet Nam in 2009, when it became one of the first countries to join the Global Antibiotic Resistance Partnership [5]. This paper considers policy recommendations made in light of that analysis and highlights existing challenges.

## Methods

**Figure 1:**
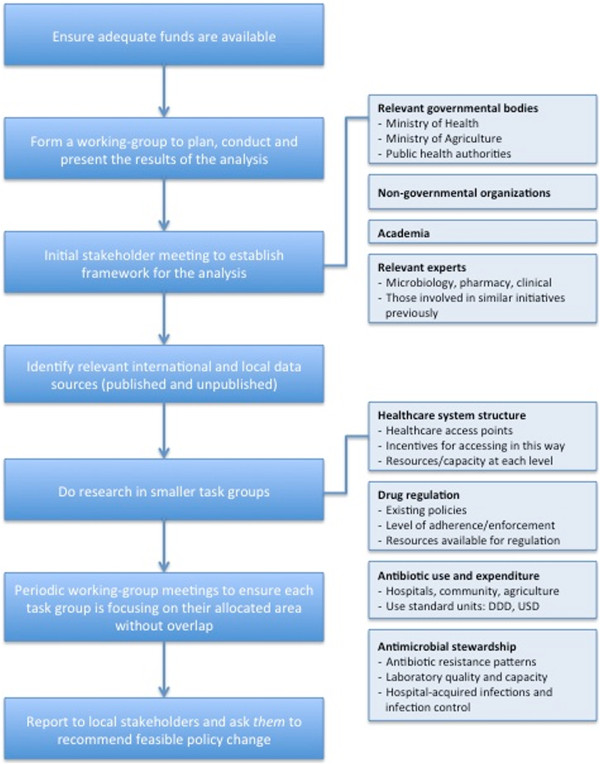
An example framework for a situation analysis in an emerging economy like Viet Nam.

We established a working-group, which included key representatives from across Viet Nam. The working-group organized a stakeholder meeting in Hanoi (September, 2009) attended by the Vietnamese Ministry of Health (MoH), WHO, hospital directors, universities, research organizations and local and international experts in public health, agriculture, microbiology, pharmacy, and clinical infectious diseases. During this and subsequent meetings, a framework for the situation analysis was outlined (Figure 1), with the primary aim of assessing and providing an overview of current patterns and determinants of antibiotic use and resistance in Viet Nam.

The analysis focused on bacterial diseases, excluding HIV, malaria and tuberculosis, and is divided into three sections: (1) healthcare system, drug regulation and supply; (2) antibiotic resistance and infection control; and (3) agricultural antibiotic use. All available sources in international and local literature since 1990 were identified, synthesized and discussed at a policy meeting.

We identified sources by searching PubMed for published papers from 1-1-1990 to 31-8-2012. The following search criteria were used: ‘antibiotic consumption’, ‘antibiotic resistance’, ‘healthcare access’, ‘drug access’, and ‘Viet Nam’. In addition, we consulted relevant unpublished reports and local Vietnamese papers and expertise. Clarification was sought from study investigators for both international and local documents when methodology or results were unclear. Information on existing policy was obtained from the MoH. Basic health indicators were obtained from the WHO, United Nations and CIA World Factbook [3,4,6].

The findings were presented at a policy workshop (June 2011) attended by local stakeholders representing the same areas of expertise as the initial stakeholder meeting. All studies were assessed for possible bias at the policy workshop before stakeholders were asked to recommend feasible policy changes to control antibiotic resistance. These recommendations were presented to the MoH to assist their formulation of a national action plan to strengthen national capacity for antimicrobial stewardship in Viet Nam.

## Results and discussion

### Healthcare system, drug regulation and supply

#### Healthcare system

Since the 1986 market reforms, several measures have improved healthcare access in Viet Nam, including health insurance schemes, enhanced infrastructure and health facilities and deregulation of drug retail [7]. Approximately 1,000 public hospitals, 100 private hospitals, 35,000 private clinics and 50,000 private pharmacies and retailers have been established [8]. In 2010 the Vietnamese Ministry of Health estimated that there were 6.52 physicians and 1.22 pharmacists per 10,000 inhabitants respectively [9].

Out-of-pocket health expenditures as a proportion of total health expenditures are estimated at 61% [10]. Self-medication is cheaper and less time-consuming than visiting a healthcare provider but often results in inappropriate drug use. One study reported average household expenditure per episode of illness for self-treatment to be 1 USD, for private providers 1.7 USD, and for public providers 4.6 USD [7]. Self-medication avoids lengthy and costly formal healthcare and is possible because prescription-only regulations are not enforced [7].

In primary care empiric antimicrobial treatment is the norm as limited resources make diagnostics difficult [11]. At present, only larger provincial and national hospitals have capacity to perform bacterial culture and antibiotic sensitivity testing. Hospitals commonly choose to provide profitable diagnostics and infectious disease diagnostics, needing expensive infrastructure, are often not profitable [12]. Inadequate laboratory quality control and assurance, high workloads and staff shortages are additional challenges, perceived as important drivers for the frequent and inappropriate antibiotic prescriptions [12].

#### Drug regulation and supply

Half of all drugs consumed in Viet Nam are made locally and more than 50% of these are antibiotics [8]. Most domestic producers are joint-stock companies, in which the State holds a 46.5% share [5]. This partnership can complicate drug regulation.

The Pharmaceutical Law (2005) made antibiotics prescription-only drugs. Despite this, antibiotics continue to be dispensed without a doctor’s prescription [13]. In 2007, the circular on Good Pharmacy Practice (GPP) was issued to improve the standard of pharmacy [14]. GPP requires pharmacies to have proper facilities, monitor drug quality, record drug consumption and comply with the prescription-only regulations. However, GPP-certified pharmacies continue to dispense antibiotics to patient’s who do not possess a doctor’s prescription [15]. Currently, there are no sanctions and as of March 2013 no pharmacy has been penalized for dispensing without prescription.

Several MoH departments issue these drug policies and are responsible for regulating private and public healthcare facilities. In 2006, 230 full-time health inspectors were employed to regulate over 39,000 pharmacy outlets [16]. Given this discrepancy of greater than 150 outlets per inspector, it is understandable that despite legislation regulation of the national drug supply chain is weak.

Drug regulation and supply is also problematic at the local level. The MoH instructs all hospitals to appoint a Drug and Therapeutics Committee (DTC). The DTC is responsible for implementing MoH guidelines, establishing lists of commonly used drugs in their hospitals and advising on rational antibiotic therapy. Certain “reserved” antibiotics, should only be dispensed after consultation with the DTC. A recent MoH instruction requires hospitals to implement local guidelines for rational and safe drug use, monitor antibiotic prescribing, strengthen hospital pharmacies and train staff to improve knowledge of drug use. However, these requirements are often not met by hospitals.

#### Patterns of antibiotic use: hospitals

In 2008–09, the most commonly sold antibiotics were oral 2^nd^ and 3^rd^ generation cephalosporins, followed by oral broad-spectrum penicillins, macrolides/azalides and fluoroquinolones. Importantly, relatively new injectable antibiotics (e.g. cephalosporins, carbapenems) are sold in hospitals, whilst older antibiotics, (e.g. chloramphenicol) predominate in retail pharmacies.

Antibiotics constitute on average 36% of hospital treatment expenditure [17]. Injectable cephalosporins have the highest sales value in hospitals and due to their high price also account for a substantial part of retail pharmacy sales (IMS Health data for Viet Nam, 2008). As the risk of resistance to older antibiotics increases it will become increasingly necessary to use newer and more expensive antibiotics, resulting in a larger proportion of hospital budgets being spent on antibiotics.

Particularly concerning is perioperative antibiotic prophylaxis [18]. Most surgeons are aware of CDC best practice guidelines [19] but lack of confidence in laboratory reporting and infection control policy motivates antibiotic overuse. Inappropriate post-operative prophylaxis has been reported in 91.8% of clean procedures and 96.3% of clean-contaminated procedures, [18] often employing combination therapy or aminoglycosides, contrary to CDC guidance. A fear of surgical site infection (SSI) likely underlies the higher rates of inappropriate prescriptions on surgical wards [20]. Recent studies show good hand hygiene is effective at reducing SSI rates [21] and adhering to CDC perioperative prophylaxis recommendations does not precipitate an increase [22].

The importance of antimicrobial stewardship is not routinely addressed in medical or pharmacy training thus knowledge and prescribing habits remain poor. An ongoing initiative led by the Vietnamese Society of Infectious Diseases aims to raise awareness of the need for sensible prescribing and improve hospital antibiotic use (http://vsid.vn/en/about-vinares.html)[23].

#### Patterns of antibiotic use: community

Mothers usually treat sick children without consulting a healthcare provider. In 1999, 91% of children in Ba Vi with symptoms of acute respiratory tract infection (ARI) were treated with broad-spectrum penicillins, 78% of them self-medicating. This corresponded to 75% of all children in that community [24]. From 1999 to 2007 *Streptococcus pneumoniae* penicillin-resistance rates increased from 8% to 75% in Ba Vi [25]. In 2007, the pattern of antibiotic use changed, as oral cephalosporins became commonly used for ARI, with similarly high proportions of patients receiving antibiotics [13].

Reasons for irrational antibiotic prescribing are the same as in other countries: perceived patient expectation; time constraints; lack of knowledge; lack of diagnostics; pressure, incentives and advertising from industry; and financial benefits for the prescriber [13]. High out-of-pocket costs of formal healthcare mean that direct purchase of drugs is more affordable for patients. A major challenge is to identify and modify the incentives for inappropriate prescribing and purchasing.

### Antibiotic resistance and infection control

Below we summarize data for key bacteria. Most data are derived from site-specific, hospital-based studies, yet the majority of illness occurs where there are few diagnostic facilities.

#### Gram-positive pathogens

From 1995 to 2000, penicillin-resistance amongst community-acquired invasive pneumococci increased from 8% to over 70% (R 71.4%; I 20.6%, n = 64) [26]. Viet Nam has the highest prevalence of penicillin- (71.4%) and erythromycin-resistance (92.1%) in Asia [26]. Resistance rates are 22 times higher in urban compared to rural children [27]. In 2009, most pneumococci were still susceptible to ceftriaxone [28]. *Streptococcus suis* is the leading cause of bacterial meningitis in adults in Viet Nam. An increase in tetracycline and chloramphenicol resistance was observed in *S. suis* over an 11-year period, associated with agricultural antibiotic use [29].

A recent study on *Staphylococcus aureus* isolates from blood showed 19% were methicillin resistant (n = 80) [30]. National surveillance of community and hospital acquired infections (HAIs) show methicillin resistance rates up to 40% [17].

#### Gram-negative pathogens

An enterobacteriaceae study in Ho Chi Minh City reported extended-spectrum beta-lactamase prevalence of 43.8% amongst hospitalized patients (n = 71) with complicated intra-abdominal infections, bloodstream infections, nosocomial pneumonia and ventilator-associated pneumonia. Prevalence has been reported to be as high as 81% amongst ICU enterobacteriaceae [31].

Over 70% of *Salmonella Typhi* isolated in a southern Viet Nam study from 1994 were multi-drug resistant (MDR) [32]. Nalidixic acid resistance increased from 4% to 97% in 12 years [33] Another study confirmed more than 80% of *S.* Typhi isolates are resistant to nalidixic acid [34] and this has been linked to treatment failure [35]. High resistance rates are also found in stool-isolated *Shigella spp.* to: trimethoprim-sulfamethoxazole, 81%; tetracycline, 74%; ciprofloxacin, 10%; and ceftriaxone, 5% [36]. Over 75% are resistant to more than one drug [37]. High rates of MDR (resistance to co-trimoxazole, naladixic acid and tetracycline) are also characteristic of *Vibrio cholerae* in Viet Nam [38] and prevalence of commensal MDR (defined as resistance to three or more antibiotics in this study) *Escherichia coli* is positively correlated with community antibiotic use [39].

Of note are the high resistance rates amongst *Helicobacter pylori*, the bacteria strongly associated with peptic ulcer disease. A study in children reported clarithromycin, metronidazole, and amoxicillin resistance rates of: 50.9%, 65.3%, and 0.5%, respectively. Importantly, clarithromycin resistance was associated with eradication failure [40].

**Figure 2:**
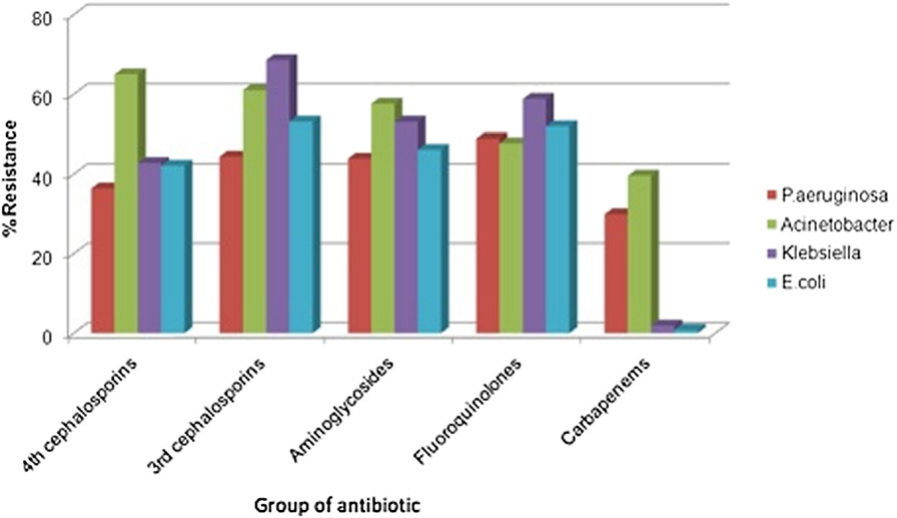
**Resistance rates for four common Gram-negative bacteria isolated from routine clinical specimens (sputum, urine, blood, pus) in hospitalized patients [**[17]**].** Source: First report on antibiotic use and resistance in Viet Nam, MoH, 2008–09.

Gram-negative bacteria producing New Delhi metallo-beta-lactamase (NDM-1) have been reported from both northern and southern Viet Nam. Furthermore these bacteria have been found in the Hanoi environment and reported in a patient admitted to a Ho Chi Minh City hospital [41,42]. High carbapenem-resistance rates are found in *Pseudomonas aeruginosa* and *Acinetobacter baumannii* HAIs (Figure 2) [31]. Most are colistin susceptible, however colistin resistance has been reported in one northern Viet Nam hospital.

#### Hospital acquired infections

In one Hanoi hospital, SSIs were associated with increased post-operative stay (8.2 days) and excess direct costs (110 USD per patient) [43]. HAIs are most common in ICUs and surgical wards. A study conducted in ICUs, surgical and obstetric departments of three district hospitals reported an overall HAI rate of 17.3%: SSIs, 55%; urinary tract infections, 21%; and lower respiratory tract infections, 17% [44]. Throughout Viet Nam, MDR Gram-negative bacteria are the most common cause of HAIs, whereas *S. aureus* is not.

#### Infection control

In 2007 most district hospitals had infection control committees but could do little to tackle issues due to limited resources. It was noted that guidelines were often outdated [11]. A recent MoH regulation on infection control in healthcare institutions, together with updated guidelines and a national health improvement project [23], represents an effort to improve capacity for infection control and make health-related activities safer for patients, staff and visitors.

Effective infection control is difficult as most Vietnamese hospitals are old, overcrowded and workloads are high. Bed occupancy can exceed 100%, especially during communicable disease outbreaks. The family is essential to care: bringing food, feeding and cleaning the patient, and this is difficult to monitor. Hospital water and solid waste is often disposed without pretreatment [45].

Viet Nam is part of the WHO clean hands campaign and more than 40% of healthcare workers know the basics of hand hygiene, however lack of facilities are reported to underlie low compliance (13.4%) [46]. Several hospitals have poor availability of water, soap and wipes. A recent study in ICUs showed hygiene precautions to be poor with fewer than 50% of patient contacts incorporating appropriate hand hygiene [47].

### Agricultural antibiotic use

Seventy percent of pharmaceuticals used in animals are antibiotics [48]. Certain antibiotics have been banned in animals since Viet Nam joined the World Trade Organization. When residues of banned antibiotics exceed set limits farms are prohibited from harvesting, processing and exporting products until the government confirms compliance. However, regulation is focused on farms producing for export, not for the domestic market.

Many antibiotics used to treat human disease are used in agriculture: *β*-lactams, aminoglycosides, macrolides, tetracyclines, (fluoro)quinolones, phenicols, pleuromutilins, lincosamides, sulfonamides, diaminopyrimidine (trimethoprim) [48]. Farmers often do not comply with regulations requiring them to stop antibiotic use before harvesting their products [48]. One study reported a positive correlation between sulfamethoxazole concentration and occurrence of sulfamethoxazole-resistant bacteria [49]. Alarmingly, polymixins (colistin) are commonly used despite not yet being registered to treat MDR infections in humans. Recently, it was found that rifampicin, a key drug for tuberculosis treatment, is being used in agriculture.

In a study performed across 4 continents, prevalence of MDR *Campylobacter* in chickens was highest in Viet Nam [50]. Sixty-six percent of *Salmonellae* in retail chicken and pork meat is MDR, with 78.4% of isolates resistant to at least one antibiotic [51]. An intervention study showed that fluctuations in bacterial resistance amongst pigs mirrored addition or withdrawal of antibiotics from their feed [52]. Recently, shrimp aquaculture has expanded and several antibiotics and antibiotic-resistant bacteria have been detected [53].

### Stakeholder recommendations from policy workshop

In June 2011 a policy workshop attended by local stakeholders representing the same areas of expertise as the initial stakeholder meeting was convened and the evidence gained from this situation analysis was discussed. Coordinated and concerted action was recognized as key to tackling antimicrobial resistance in Viet Nam and other emerging economies. This action must be carefully considered to minimize possible adverse health impacts.

A better understanding of appropriate antimicrobial use must be fostered amongst clinicians. The development of a national antibiotic stewardship program is central to this. Surveillance programs that specifically monitor antimicrobial use in hospitals, the community and agriculture need to be established. Public educational initiatives should be considered and have potential for great impact given the high adult literacy rate of 93.4%. It was agreed that adequate legislation already exists to control antimicrobial resistance in Viet Nam but there is an urgent need to prioritize effective enforcement and imposition of appropriate penalties (Table 2). Better mapping of the incentive structure for manufacturers, providers and purchasers of antibiotics will likely improve compliance with existing legislation. The main priorities from the policy workshop are summarized below.

**Table 2:** Current policies related to antibiotic use and problems with their implementation

**Policy area**	**Problems with implementation**	**Recommendations**
Antibiotics are prescription only drugs	Discrepancy between number of regulators and retail outlets. No sanctions or penalties for non-compliance.	Allocate more resources to implement the law. Ensure appropriate sanctions and penalties are enforced for non-compliant providers. Public educational initiatives to raise awareness and take advantage of the high literacy rate.
Hospitals need a Drug ad Therapeutics Committee (DTC)	DTCs are not trained in antimicrobial stewardship methodologies and do not have access to current resistance data. Many hospitals do not have DTCs.	Provide tools/guidance on effective hospital antibiotic stewardship. Provide them with reliable and up-to-date resistance data.
Hospitals need an infection control committee	Standardized surveillance structures are not in place. Many hospitals do not have infection control committees.	Provide infection control committees with funding to carry out their activities and improve infrastructure. Train them to use standardized surveillance methodologies and indicators to monitor progress, such as HAI rates by department and hand washing compliance.
Laboratory enhancement program	Inappropriate resistance testing gives erroneous and discrepant results	Ensure quality of laboratory testing by issuing national testing guidelines, including quality control strains. Consider setting up a national reference center (center of excellence) for antibiotic resistance testing and overseeing external quality control. Create an interactive network for sharing information (data, guidelines, expertise).
National antibiotic resistance surveillance	Lack of communication between institutions regarding resistance data	Pool data from all hospitals to create a national resistance database. Release a national annual report that includes both antibiotic use and resistance data in the same document.
Hospital antibiotic use surveillance	No standardized reporting of antibiotic consumption data	Standardize antibiotic usage indicators to international units, like Defined Daily Dosage (DDD) per 100 bed days. Release a national annual report that includes both antibiotic use and resistance data in the same document.
Medical and pharmacy school curriculum	Insufficient emphasis placed on antimicrobial stewardship	Provide sufficient time to teach and train students on appropriate antibiotic use and resistance. Highlight the growing public health problem of drug resistance.
Standard treatment guidelines	Current treatment guidelines are outdated	Ensure timely and evidence based updates of treatment guidelines for infectious diseases, utilizing local resistance data.
Pharmacovigilance	Adequate funding and resources	Engage the center for pharmacovigilance in tackling the issue of inappropriate antibiotic prescribing.
Stop agricultural antibiotic use before harvesting products	Enforcement focuses on farmers producing for export	Enforce the law. Set up a similar national antibiotic use and resistance surveillance system for the agricultural industry. Compile a national annual report that includes both antibiotic use and resistance data in the same document.

List of current policies related to antibiotic use and resistance, problems with their implementation and recommendations to improve implementation.

### Policy priorities to control antibiotic resistance

1. Enforcement of existing regulations involving antibiotic use in hospitals, the community and agriculture with stronger regulatory capacity and monitoring. The imposition of penalties in a more effective way should be considered.

2. Develop an antibiotic stewardship program (ASP) with an ASP steering committee that includes standard treatment guideline (STG) development, implementation, regular updates, and audits. STGs need to be evidence based and use local epidemiology and resistance rates.

3. Create three national surveillance programs and create the required national reference laboratories to ensure the availability and quality of testing for the surveillance program. The three national surveillance programs are:

• Hospital antibiotic use and resistance (conducted by MoH)

• Community antibiotic use and resistance (conducted by NIHE)

• Antibiotic use, resistance and residue in animals (conducted by MARD).

4. Improve access to non-medicated animal feed for farmers [54]. It is recommended to limit the use of colistin in agriculture, as it is a last resort drug for MDR infections in humans.

5. Improve education regarding antibiotic use and resistance for those working in the healthcare system and agriculture.

6. The MoH and MARD are recommended to develop a joint action plan. During its development, stakeholders (governmental agencies, WHO, FAO, hospitals, pharmacies, industry and academia) should be consulted.

NIHE = National Institute for Hygiene and Epidemiology.

MARD = Ministry of Agriculture and Rural Development.

FAO = Food and Agriculture Organization of the United Nations.

Policy priorities proposed by local stakeholders during a policy workshop in June 2011 which has assisted the MoH with formulation of a national action plan to improve national capacity for antimicrobial stewardship in Viet Nam. Many of these priorities may be applicable to other emerging economies tackling the growing threat of antimicrobial resistance.

### Limitations

#### Availability of data

Formulating policy to tackle antimicrobial resistance requires a broad analysis of a country and its health system to better understand the patterns and determinants that drive antibiotic use and resistance. To do this it is essential to consult local data, however this can be of poor quality, inadequate or absent. For example whilst substandard or counterfeit antibiotics (except antimalarials) are not recognized as a particular problem in Viet Nam, there is insufficient data to draw reliable conclusions with respect to drug quality.

We found that it was necessary to carefully examine the data and investigate the reliability of the sources before drawing conclusions upon which we could make credible policy recommendations. Identifying areas of sub-optimal data is important as it can motivate improvement. High quality local data is essential to allow effective interventions to be designed and appropriately targeted.

#### Mapping of the incentive structure

Effective recommendations to alter antibiotic use must consider the incentives for manufacturers, providers and purchasers. Mapping and addressing of the incentive structures is difficult and requires long-term cooperation between local stakeholders and policy makers. Regular review of any intervention is necessary to assess whether it has been successful in modifying these incentives and changing behavior.

## Conclusions

Increased access to antibiotics has contributed to improved health outcomes in Viet Nam. However antibiotic overuse in hospitals, the community and agriculture has promoted rapid development of bacterial resistance, eroding the health asset antimicrobials constitute. As a result Viet Nam, like many emerging economies, faces a considerable challenge in attempting to control antimicrobial resistance.

Antimicrobial resistance may represent the greatest global threat by an Emerging Infectious Disease issue. Its insidious nature may not have the cache of SARS, pandemic influenza or Ebola but its impact on public health is likely to be far greater. Like other Emerging Infectious Diseases, drug resistance can quickly spread from one country to another and hence the need for concerted national and international action.

There is consensus amongst the political and medical leadership of Viet Nam that antibiotic resistance is a serious and growing problem. The emergence and spread of carbapenem-resistant Gram-negative bacteria, a phenomenon not unique to Viet Nam, illustrates this. High resistance rates mean that many antibiotic regimens in current treatment guidelines are already ineffective across a broad range of clinical syndromes and pathogens. There are many examples of legislation and initiatives designed to limit unnecessary antibiotic use (Table 2). However, judged against their intended goal, these policies have been ineffective.

High individual out-of-pocket expenditure may have increased consumer control and weakened the influence of the State on the healthcare market. Policies to reduce out-of pocket expenditure through health insurance may reduce the ability of individuals to shape the healthcare market and be important in revitalizing the influence of the State [55]. Point-of-care tests to improve diagnostics in primary healthcare may reduce antibiotic overuse. These and other policy options need careful consideration for their feasibility, affordability and possible adverse impacts.

There are many barriers preventing effective enforcement of regulations designed to improve antimicrobial stewardship in developing countries: insufficient funding and lack of expertise, human resources and financial incentives. It is important to foresee possible negative consequences of enforcement: financial losses and reduced healthcare access. Accounting for this will improve policy implementation in these settings. Furthermore, it is important to know where to focus regulatory efforts: a focal point too far downstream may create targets too numerous or dispersed for enforcement to be feasible.

Patients, physicians, veterinarians, clinics and hospitals, and retailers – from large pharmacies to local drug sellers – have little motivation to weigh the negative impact of their antibiotic use on others, especially those in the future. Policy solutions must alter incentives for patients, physicians and others in the healthcare system to act in society’s best interests. Better quantification and mapping of the incentive structure for antibiotic prescribing in emerging economies would support the design of more effective interventions.

Antibiotic resistance does not yet top any list of national healthcare problems for Viet Nam or other emerging economies. Antibiotic stewardship should not drain resources from more pressing concerns. Done correctly, controlling antibiotic resistance should either be cost neutral or one of the few health interventions that saves money.

Recently the MoH in Viet Nam has approved a national action plan to control antibiotic resistance, which includes development of systematic surveillance structures to monitor antibiotic use and resistance, and laboratory participation in quality assurance schemes. However, it is not yet known what budget will be made available to implement this national action plan. Part of this analysis has provided important input to this initiative, illustrating that with relatively little effort and funds, a lot can be achieved on a national scale. We believe it can be a useful framework for similar initiatives in other countries, and emerging economies in particular.

## Competing interests

The authors’ declare that they have no competing interests.

## Authors’ contributions

Conception and design: All authors, Data collection and analysis: All authors, Drafting of manuscript: KVN, NTTD, AC, HFLW, Final editing and approval: All authors.

## Pre-publication history

The pre-publication history for this paper can be accessed here:

http://www.biomedcentral.com/1471-2458/13/1158/prepub
